# Associations of specific types of physical activities with 10-year risk of cardiovascular disease among adults: Data from the national health and nutrition examination survey 1999–2006

**DOI:** 10.3389/fpubh.2022.964862

**Published:** 2022-07-25

**Authors:** Bingsen Huang, Qian Wang, Xin Wang, Lei Wang, Peiyao Ma, Fengling Wang, Changchun Du

**Affiliations:** ^1^Department of Cardiology, Henan Provincial Chest Hospital, Zhengzhou, China; ^2^Women and Infants Hospital of Zhengzhou, Zhengzhou, China

**Keywords:** physical activities, cardiovascular disease risk, specific types, synergistic effect, antagonistic effects

## Abstract

**Background:**

Physical activity plays a key role in the prevention of cardiovascular disease (CVD). However, previous studies focused predominantly on the associations of the total amount of physical activity with CVD. There were few evidences on the associations of specific sport disciplines with CVD. Furthermore, little was known on the interactions between the different types of sports on CVD risk. Therefore, this study aimed to examine the independent associations of specific types of physical activities with the 10-year risk of CVD, and further evaluate the interactions between specific types of physical activities on the 10-year risk of CVD in US adults.

**Methods:**

This study used the data of the National Health and Nutrition Examination Survey (NHANES) 1999-2006. Participants aged ≥ 30 years and with free of CVD were eligible. The physical activity questionnaire is used to collect general information on leisure-time activities in the past 30 days, including the frequency, duration, and intensity of participation in each activity. The exposures of interest included cycling, swimming, aerobics, running, American Football, basketball, and racquet sports. The Framingham risk score algorithm was used to assess 10-year CVD risk based on age, high density lipoprotein, total cholesterol, systolic blood pressure, smoking status, and diabetes. A higher total score reflects a greater risk of CVD.

**Results:**

This study included 10829 participants. Compared to no participation, participation in cycling (β = −0.890, *95% CI*:−1.278,−0.502, *P* < 0.001), running (β = −1.466, *95% CI*:−1.837,−1.095, *P* < 0.001), American Football (β = −2.934, *95% CI*:−3.750,−2.119, *P* < 0.001), basketball (β = −1.968, *95% CI*:−2.645,−1.291, *P* < 0.001), and aerobics (β = −0.980, *95% CI*:−1.352,−0.608, *P* < 0.001) was associated with a lower CVD risk. Furthermore, cycling was antagonistic with basketball and racquet sports in the associations with CVD risk. An antagonistic action between swimming and aerobics was also observed. Nevertheless, running was synergistic with cycling, aerobics, and racquet sports in the associations with CVD risk.

**Conclusions:**

There were inverse associations of specific types of physical activities with CVD risk. Furthermore, there might be synergistic and antagonistic associations of multiple types of physical activities with CVD risk.

## Introduction

Cardiovascular disease (CVD) has been the leading cause of mortality in the general population worldwide (1). Primordial prevention has been acknowledged as an important population-wide approach for preventing the development and progression of CVD (2, 3). Physical activity (PA) is considered as a cornerstone for CVD prevention (4). Insufficient PA is a key risk factor for CVD and death (5, 6). According to the Global Burden of Diseases report, there were 2.1 million premature deaths and 4.5 million disability-adjusted life-years worldwide attributed to insufficient PA in 2013 (7). It is estimated that one third or more of adults worldwide and approximate 50% of adults in US do not reach the recommended amount of moderate or vigorous-intensity activity (8, 9). Given the decreasing level of PA worldwide in recent decades, it is essential to investigate the association of PA with the risk of CVD.

There were many studies to examine the associations of PA with health outcomes, including mortality, CVD, hypertension, metabolic syndrome, and obesity (10–13). However, most of the previous studies limited to the associations of the total amount of physical activity with CVD (13). There were few evidences on the nature and scope of the associations of specific sport disciplines with CVD. Furthermore, little was known on the interactions between the different types of sports on the risk of CVD. Previous study reported that leisure-time PA contributes more to the reduction of CVD risk than other types of activity (14). Therefore, a cross-sectional study using the data of the National Health and Nutrition Examination Survey (NHANES) was conducted to assess the associations of specific types of sports and exercises in leisure time with the risk of CVD in this study. The main aims of the present study were to examine the independent associations of specific types of physical activities and the intensity, duration, and volume of sports with the 10-year risk of CVD, and further evaluate the interactions between specific types of physical activities on the 10-year risk of CVD in US adults.

## Materials and methods

### Study design

Data analyzed in this study were derived from the NHANES. As a repeated cross-sectional survey, the NHANES aims to assess and supervise the health and nutritional status in the US population. A stratified, multistage random procedure is used to recruit sample from the US population. The major information collected in the NHANES include demographic, socioeconomic, dietary, health-related behaviors, physiological measurements, and laboratory tests. The details of the NHANES are provided on the website of the Centers for Disease Control and Prevention (https://www.cdc.gov/). Given specific leisure- time activities are collected only in the continuous NHANES 1999–2000, 2001–2002, 2003–2004, and 200–2006, this study only used the data of the NHANES from 1999 to 2006.

### Study population

**Figure 1:**
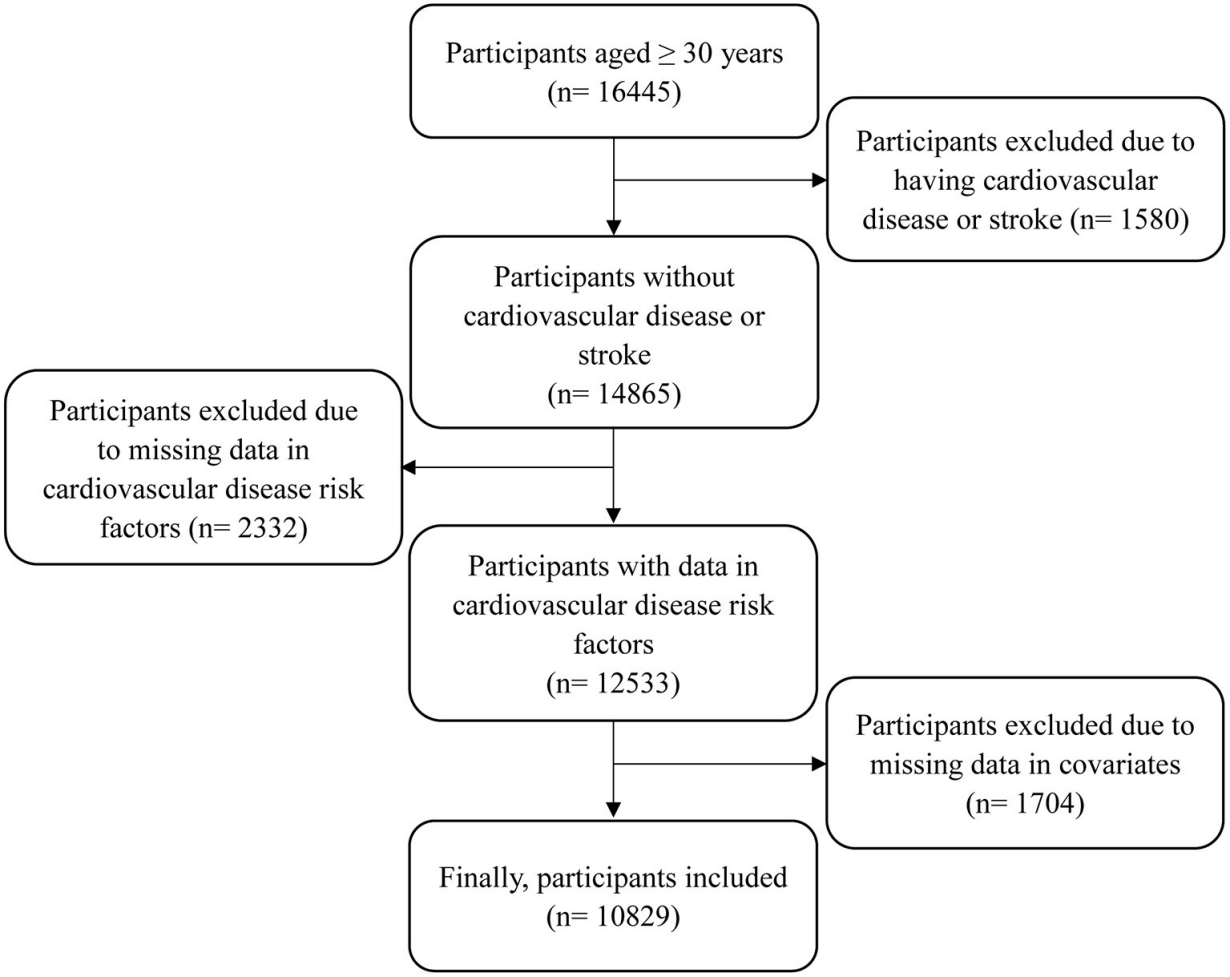
The flow chart of participants included.

The 10-year CVD risk was assessed using the Framingham Risk Score (FRS), which focuses on adults aged 30 year or older. Therefore, participants aged ≥ 30 years and with free of CVD were eligible in this study. The detailed inclusion criteria included: participants having complete data of specific leisure- time activities; participants having complete data of CVD risk factors, including age, high density lipoprotein (HDL) cholesterol, total cholesterol (TC), systolic blood pressure (SBP) and treatment for hypertension, smoking status, and history of diabetes; and participants having complete data of main covariates, such as physical measurements and health-related indicators. The detailed exclusion criteria included: participants having cardiovascular disease or stroke; and participants having missing data of analyzed variables. The detailed process is shown in Figure 1. This study was approved by the NHANES Institutional Review Board (1999-2002: Protocol #98-12). Written consent was obtained from participants.

### Physical activity assessment

The physical activity questionnaire (PAQ) is used to collect general information on leisure-time activities in the past 30 days. If respondents participated in leisure-time activities, the frequency, duration, and intensity of participation in specific types of physical activities were inquired. The corresponding questions included as follows: for frequency: Over the past 30 day, how often did you do [activity name]?; for duration: Over the past 30 days, on average about how long did you do [activity name]?; and for perceived relative intensity: What do you think is the intensity level of [activity name]?. The overall participation was divided into “none” or “any.” The duration per month was divided into “none,” “low,” and “high” using the sex-specific medians as shown in Supplementary Table 1. The relative perceived intensity was divided into “none,” “moderate,” and “vigorous.” The intensity-weighted volume per month [metabolic equivalent (MET)] was calculated using the PA Compendium, and was divided into “none,” “low,” and “high” using the sex-specific medians as shown in Supplementary Table 1 (15). The exposures of interest in this study included cycling, swimming, aerobics including aerobics, dance, martial arts, yoga, and gymnastics, running including jogging, running, and treadmill, American Football including football and soccer, basketball, and racquet sports including baseball, golf, hockey, racquetball, softball, and tennis. Other sports were not included in this study because of the low participation rates.

### Measurement

Serum TC and HDL levels were assayed using Hitachi 704 Analyzer in Lipoprotein Analytical Laboratory at the Johns Hopkins University School of Medicine. Plasma glucose level was assayed using Enzyme hexokinase in the Department of Child Health at the University of Missouri-Columbia. SBP and diastolic blood pressure (DBP) were measured three times using a mercury sphygmomanometer, and the average measurements were used to analysis. A question of “Because of your hypertension, have you ever taken prescribed medicine?” was used to identify whether treatment for hypertension. Similarly, a question was used to identify the history of diabetes as follows: Other than during pregnancy, have you ever been told by a doctor or health professional that you have diabetes or sugar diabetes? Participants having the history of diabetes, fasting plasma glucose≥ 126 mg/dL, or use of anti-diabetic medications were considered to have diabetes (16). Current smoking status was assessed at the beginning of the study: Do you smoke cigarettes now?

### Assessment of CVD risk

The sex-specific FRS algorithm based on the original Framingham Heart Study was used to assess the 10-year CVD risk (17). The FRS algorithm includes six cardiometabolic factors as age, HDL, TC, SBP dependent of treatment, smoking status, and diabetes. Age is divided into 10 groups by interval of 5 years as 30–34, 35–39, 40–44, 45–49, 50–54, 55–59, 60–64, 65–69, 70–74, and ≥75 years, which are assigned to scores 0, 2, 5, 6, 8, 10, 11, 12, 14, and 15 for males, and scores 0, 2, 4, 5, 7, 8, 9, 10, 11, and 12 for females. HDL is divided into 5 groups as follows: <35 mg/dL, 35–44 mg/dL, 45–49 mg/dL, 50–59 mg/dL, and ≥60 mg/dL, which are assigned to scores 2, 1, 0,−1, and−2 for males and females. TC is also divided into 5 groups: <160 mg/dL, 160–199 mg/dL, 200–239 mg/dL, 240–279 mg/dL, and ≥280 mg/dL, which are assigned to scores 0, 1, 2, 3, and 4 for males, and scores 0, 1, 3, 4, and 5 for females. For males, SBP is divided into 5 groups: <120 mmHg, 120–129 mmHg, 130–139 mmHg, 140–159 mmHg, and ≥160 mmHg, which are assigned to scores−2, 0, 1, 2, and 3 if not treatment, and scores 0, 2, 3, 4, and 5 if treatment. For females, SBP is divided into 6 groups: <120 mmHg, 120–129 mmHg, 130–139 mmHg, 140–149 mmHg, 150–159 mmHg, and ≥160 mmHg, which are assigned to scores−3, 0, 1, 2, 4, and 5 if not treatment, and scores−1, 2, 3, 5, 6, and 7 if treatment. If smoking, scores are assigned to 4 for males and 3 for females, but if not smoking, scores are assigned to 0 for males and females. Similarly, if having diabetes, scores are assigned to 3 for males and 4 for females, but if not having diabetes, scores are assigned to 0 for males and females. Total score is calculated as the sum of points of all cardiometabolic factors. A higher total score indicates a higher risk of CVD. The FRS algorithm has been validated in other studies (18, 19).

### Statistical analysis

Normality of continuous variables was tested using *Kolmogorov-Smirnov test*. Continuous data with normal distribution are presented as means ± standard deviations (SDs) and are compared between male and female using *t-test*. The duration and volume of specific types of physical activities are presented as medians [interquartile ranges (IQRs)]. Categorized variables are expressed as frequencies (percentages) and are compared between-group using *chi-square test*. General linear regression model was employed to examine the associations of specific types of physical activities and the intensity, duration, and volume of sports with the 10-year risk of CVD, and evaluate the interactions between specific types of physical activities on the 10-year risk of CVD. Furthermore, the sampling weight provided in the NHANES dataset was taken into account to adjust for non-responses bias and over-sampling of certain populations using PROC SURVEYREG in SAS 9.4. In all models, sex, BMI, race, current alcohol consumption, annual household income, intakes of energy, protein, carbohydrate, and fat, and the volume of other physical activity (MET, excluding the volume of the sport that was the main exposure in the corresponding model) were adjusted. In order to assess the influence of missing data, all variables with missing data were imputed for five times using R package of “*mice*.” A sensitivity analysis was conducted to examine the associations of specific types of physical activities with CVD risk using the complete dataset. All analyses were conducted using SAS 9.4 (SAS Institute Inc., Cary, NC, USA.). A two-tailed *P* ≤ 0.05 was considered to be statistically significant.

## Results

### Characteristics of participants

There were 10,829 participants in the final analysis. The average of age was 53.46 ± 15.67 years. The average of CVD risk score was 10.13 ± 6.94. There were significant differences between male and female in all characteristics, except age (*P* = 0.186), race (*P* = 0.133), history of diabetes (*P* = 0.215), SBP (*P* = 0.100), and swimming (*P* = 0.235). Compared to females, males were more likely to participate in cycling, running, American Football, basketball, and racquet sports, and were less likely to participate in aerobics. Meanwhile, males had a more CVD risk score than females (*P* < 0.001) as shown in Table 1.

**Table 1:** Characteristics of all participants by sex.

**Characteristics**	**Total sample** **(*****n*** = **10,829)**	**Male** **(*****n*** = **5,267)**	**Female** **(*****n*** = **5,562)**	***P*** **for sex**
Age (mean ± SD, years)*	53.46 ± 15.67	53.66 ± 15.41	53.26 ± 15.90	0.186
BMI (mean ± SD, kg/m^2^) *	28.67 ± 6.16	28.26 ± 5.37	29.06 ± 6.81	<0.001
Race [*n* (%)]^#^				0.133
Non-Hispanic white	5,794 (53.50)	2,846 (54.03)	2,948 (53.00)	
Non-Hispanic black	2,006 (18.52)	981 (18.63)	1,025 (18.43)	
Mexican-American	2,243 (20.71)	1,089 (20.68)	1,154 (20.75)	
Other	786 (7.26)	351 (6.66)	435 (7.82)	
Current smoking [*n* (%)]^#^				<0.001
No	8,584 (79.27)	3,974 (75.45)	4,610 (82.88)	
Yes	2,245 (20.73)	1,293 (24.55)	952 (17.12)	
Current alcohol consumption [*n* (%)]^#^				<0.001
No	5,524 (51.01)	2,195 (41.67)	3,329 (59.85)	
Yes	5,305 (48.99)	3,072 (58.33)	2,233 (40.15)	
Annual household income [*n* (%)]^#^				<0.001
< $20,000	2,422 (22.37)	1,056 (20.05)	1,366 (24.56)	
$20,000~45,000	3,478 (32.12)	1,692 (32.12)	1,786 (32.11)	
≥$45,000	4,929 (45.52)	2,519 (47.83)	2,410 (43.33)	
History of diabetes [*n* (%)]^#^				0.215
No	9,730 (89.85)	4,713 (89.48)	5,017 (90.20)	
Yes	1,099 (10.15)	554 (10.52)	545 (9.80)	
Intake of energy (mean ± SD, kcal/day)*	2100.03 ± 983.66	2447.52 ± 1082.54	1770.97 ± 742.72	<0.001
Intake of protein (mean ± SD, gm/day)*	79.99 ± 41.50	93.26 ± 45.84	67.43 ± 32.24	<0.001
Intake of carbohydrate (mean ± SD, gm/day)*	257.23 ± 126.07	293.16 ± 138.80	223.20 ± 101.59	<0.001
Intake of fat (mean ± SD, gm/day)*	79.13 ± 45.82	91.80 ± 51.34	67.12 ± 36.00	<0.001
SBP (mean ± SD, mmHg)*	127.21 ± 20.46	127.54 ± 17.88	126.89 ± 22.63	0.100
DBP (mean ± SD, mmHg)*	71.66 ± 13.64	73.20 ± 13.74	70.20 ± 13.38	<0.001
Plasma glucose (mean ± SD, mg/dL) *	99.89 ± 35.74	102.12 ± 37.07	97.77 ± 34.31	<0.001
TC (mean ± SD, mg/dL) *	206.87 ± 42.40	204.43 ± 43.08	209.17 ± 41.62	<0.001
HDL-cholesterol (mean ± SD, mg/dL) *	53.43 ± 16.28	47.89 ± 13.77	58.68 ± 16.73	<0.001
Cardiovascular disease risk score (mean ± SD) *	10.13 ± 6.94	11.31 ± 6.33	9.01 ± 7.29	<0.001
Cycling [*n* (%)]^#^				<0.001
None	9,765 (90.17)	4,640 (88.10)	5,125 (92.14)	
Any	1,064 (9.83)	627 (11.90)	437 (7.86)	
Swimming [*n* (%)]^#^				0.235
None	10,359 (95.66)	5,051 (95.90)	5,308 (95.43)	
Any	470 (4.34)	216 (4.10)	254 (4.57)	
Running [*n* (%)]^#^				<0.001
None	9,473 (87.48)	4,526 (85.93)	4,947 (88.94)	
Any	1,356 (12.52)	741 (14.07)	615 (11.06)	
American Football [*n* (%)]^#^				<0.001
None	10,637 (98.23)	5,103 (96.89)	5,534 (99.50)	
Any	192 (1.77)	164 (3.11)	28 (0.50)	
Basketball [*n* (%)]^#^				<0.001
None	10,479 (96.77)	4,971 (94.38)	5,508 (99.03)	
Any	350 (3.23)	296 (5.62)	54 (0.97)	
Racquet sports [*n* (%)]^#^				<0.001
None	10,065 (92.94)	4,684 (88.93)	5,381 (96.75)	
Any	764 (7.06)	583 (11.07)	181 (3.25)	
Aerobics [*n* (%)]^#^				<0.001
None	9,413 (86.92)	4,769 (90.54)	4,644 (83.50)	
Any	1,416 (13.08)	498(9.46)	918(16.50)	

BMI, body mass index; HDL, high density lipoprotein; TC, total cholesterol; SBP, systolic blood pressure; DBP, diastolic blood pressure. * These variables were analyzed using t-test. ^#^These variables were analyzed using chi-square.

### Associations of specific types of physical activities with CVD risk

Table 2 shows the associations of specific types of physical activities with CVD risk. When analyzing separately the associations of specific types of PA with CVD risk, significant inverse associations with CVD risk were observed in cycling, running, American Football, basketball, and aerobics (all *P* < 0.001). Furthermore, participation in American Football will result in a decrease of approximate 3 points in CVD risk score, which was the strongest among all types of physical activities. However, no significant associations of swimming (*P* = 0.096) and racquet sports (*P* = 0.164) with CVD risk were observed.

**Table 2:** Associations of specific types of physical activities participation with CVD risk in adults aged ≥ 30 years^*^.

	β	***95% CI***	***P-*** **value**
Cycling
None	*Ref*		
Any	−0.890	−1.278,−0.502	<0.001
Swimming
None	*Ref*		
Any	−0.458	−0.998, 0.081	0.096
Running
None	*Ref*		
Any	−1.466	−1.837,−1.095	<0.001
American football
None	*Ref*		
Any	−2.934	−3.750,−2.119	<0.001
Basketball
None	*Ref*		
Any	−1.968	−2.645,−1.291	<0.001
Racquet sports
None	*Ref*		
Any	−0.312	−0.751, 0.128	0.164
Aerobics
None	*Ref*		
Any	−0.980	−1.352,−0.608	<0.001

* Sex, BMI, race, current alcohol consumption, annual household income, intakes of energy, protein, carbohydrate, and fat, and the volume of other physical activity (MET, excluding the volume of the sport that was the main exposure in the corresponding model) were adjusted.

### Associations of the intensity of specific types of physical activities with CVD risk

Table 3 shows that participation in specific types of physical activities any intensity was associated with CVD risk, except racquet sports (*P* for trend = 0.155). Furthermore, vigorous intensity of participation in cycling, swimming, running, American Football, and aerobics was more strongly associated with CVD risk than moderate intensity. Similarly, the strongest association of American Football with CVD risk was observed (β = −2.310 and−3.237) among all types of physical activities despite of moderate or vigorous intensity.

**Table 3:** Associations of the intensity of specific types of physical activities with CVD risk in adults aged ≥ 30 years^*^.

	β	***95% CI***	***P-*** **value**
Cycling
None	*Ref*		
Moderate	−0.505	−1.125, 0.116	0.111
Vigorous	−1.089	−1.557,−0.621	<0.001
*P* for trend			<0.001
Swimming
None	*Ref*		
Moderate	0.008	−0.780, 0.797	0.984
Vigorous	−0.807	−1.512,−0.103	0.025
*P* for trend			0.041
Running
None	*Ref*		
Moderate	0.790	−0.075, 1.655	0.073
Vigorous	−1.977	−2.366,−1.588	<0.001
*P* for trend			<0.001
American football
None	*Ref*		
Moderate	−2.310	−3.693,−0.926	0.001
Vigorous	−3.237	−4.223,−2.251	<0.001
*P* for trend			<0.001
Basketball
None	*Ref*		
Moderate	−2.016	−3.249,−0.782	0.001
Vigorous	−1.949	−2.746,−1.152	<0.001
*P* for trend			<0.001
Racquet sports
None	*Ref*		
Moderate	−0.266	−0.759, 0.227	0.291
Vigorous	−0.447	−1.283, 0.388	0.294
*P* for trend			0.155
Aerobics
None	*Ref*		
Moderate	−0.335	−0.809, 0.1380	0.165
Vigorous	−1.792	−2.312,−1.271	<0.001
*P* for trend			<0.001

* Sex, BMI, race, current alcohol consumption, annual household income, intakes of energy, protein, carbohydrate, and fat, and the volume of other physical activity (MET, excluding the volume of the sport that was the main exposure in the corresponding model) were adjusted.

### Associations of the duration of specific types of physical activities with CVD risk

Table 4 displays that participation in specific types of physical activities any duration was associated with CVD risk, except swimming (*P* for trend = 0.321) and racquet sports (*P* for trend = 0.155). Furthermore, higher duration of participation in running, American Football, basketball, and aerobics was associated with a lower CVD risk. However, compared to no participation in racquet sports, low duration of participation was associated with a lower CVD risk (β = −1.337, *95% CI*:−1.916,−0.758, *P* < 0.001), but high duration of participation was associated with a higher CVD risk (β = 0.761, *95% CI*: 0.172, 1.351, *P* = 0.011).

**Table 4:** Associations of the duration of specific types of physical activities with CVD risk in adults aged ≥ 30 years^*^.

	β	***95% CI***	***P-*** **value**
Cycling
None	*Ref*		
Low	−1.331	−1.864,−0.798	<0.001
High	−0.480	−0.986,−0.026	0.063
*P* for trend			0.001
Swimming
None	*Ref*		
Low	−0.906	−1.620,−0.193	0.013
High	−0.007	−0.778, 0.765	0.987
*P* for trend			0.321
Running
None	*Ref*		
Low	−1.365	−1.872,−0.858	<0.001
High	−1.566	−2.037,−1.094	<0.001
*P* for trend			<0.001
American football
None	*Ref*		
Low	−2.847	−3.872,−1.822	<0.001
High	−3.017	−4.253,−1.781	<0.001
*P* for trend			<0.001
Basketball
None	*Ref*		
Low	−1.508	−2.450,−0.566	0.002
High	−2.466	−3.386,−1.546	<0.001
*P* for trend			<0.001
Racquet sports
None	*Ref*		
Low	−1.337	−1.916,−0.758	<0.001
High	0.761	0.172, 1.351	0.011
*P* for trend			0.636
Aerobics
None	*Ref*		
Low	−0.842	−1.340,−0.344	0.001
High	−1.110	−1.609,−0.611	<0.001
*P* for trend			<0.001

* Sex, BMI, race, current alcohol consumption, annual household income, intakes of energy, protein, carbohydrate, and fat, and the volume of other physical activity (MET, excluding the volume of the sport that was the main exposure in the corresponding model) were adjusted.

### Associations of the volume of specific types of physical activities with CVD risk

Significant linear trends across the volume of none, low, and high were observed in all types of physical activities, except racquet sports (*P* = 0.088). In these types of physical activities, CVD risk decreased with an increase in the volume of participation. Furthermore, higher volume of participation in all types of sports, except basketball and racquet sports, was associated with a lower CVD risk. Consistent with that mentioned above, participation in American Football was the most effective sport to reduce the risk of CVD despite of low or high volume (β = −2.731 and−3.099) as shown in Table 5.

**Table 5:** Associations of the volume of specific types of physical activities with CVD risk in adults aged ≥ 30 years^*^.

	β	***95% CI***	***P-*** **value**
Cycling
None	*Ref*		
Low	−0.505	−1.125, 0.116	0.111
High	−1.089	−1.557,−0.621	<0.001
*P* for trend			<0.001
Swimming
None	*Ref*		
Low	0.617	−0.722, 1.957	0.367
High	−0.679	−1.257,−0.101	0.021
*P* for trend			0.042
Running
None	*Ref*		
Low	0.878	−0.029, 1.784	0.058
High	−1.956	−2.344,−1.569	<0.001
*P* for trend			<0.001
American football
None	*Ref*		
Low	−2.731	−3.869,−1.593	<0.001
High	−3.099	−4.230,−1.967	<0.001
*P* for trend			<0.001
Basketball
None	*Ref*		
Low	−2.016	−3.249,−0.782	0.001
High	−1.949	−2.746,−1.152	<0.001
*P* for trend			<0.001
Racquet sports
None	*Ref*		
Low	0.714	−0.480, 1.907	0.241
High	−0.465	−0.932, 0.002	0.051
*P* for trend			0.088
Aerobics
None	*Ref*		
Low	−0.853	−1.493,−0.214	0.009
High	−1.030	−1.458,−0.601	<0.001
P for trend			<0.001

* Sex, BMI, race, current alcohol consumption, annual household income, intakes of energy, protein, carbohydrate, and fat, and the volume of other physical activity (MET, excluding the volume of the sport that was the main exposure in the corresponding model) were adjusted.

### The interactions between the different types of physical activities on the risk of CVD

Supplementary Table 2 shows that significant interactions were observed between cycling and running (*P* = 0.016), between cycling and basketball (*P* = 0.002), between cycling and racquet sports (*P* = 0.002), between swimming and aerobics (*P* = 0.041), between running and American Football (*P* = 0.009), between running and basketball (*P* = 0.044), and between running and aerobics (*P* = 0.019). Further analysis was then conducted by forming four groups combining interactional two types of sports. Table 6 shows that group with both participation in cycling and basketball was not associated with CVD risk compared to the group with both no participation (β = −0.555, *95% CI*:−1.870, 0.761, *P* = 0.409). The similar phenomenon was observed in the combinations of cycling and racquet sports (β = −0.124, *95% CI*:−0.930, 0.682, *P* = 0.763) as well as swimming and aerobics (β = −0.686, *95% CI*:−1.720, 0.347, *P* = 0.193). Therefore, it indicates that there might be antagonistic effects between cycling and basketball, between cycling and racquet sports, and between swimming and aerobics on the risk of CVD. However, groups with both participation in running and cycling, running and aerobics, and running and racquet sports had greater absolute values of regression coefficient than the groups with only participation in one sport, indicating the presence of significant synergistic associations of running and cycling, running and aerobics, and running and racquet sports with CVD risk.

**Table 6:** The interactions between different types of physical activities on CVD risk in adults aged ≥ 30 years^*^.

		β	***95% CI***	***P-*** **value**
Cycling	Running			
None	None	*Ref*		
Any	None	−1.733	−2.155,−1.311	<0.001
None	Any	−1.228	−1.693,−0.762	<0.001
Any	Any	−1.808	−2.422,−1.195	<0.001
Cycling	Basketball			
None	None	*Ref*		
Any	None	−2.512	−3.263,−1.761	<0.001
None	Any	−1.067	−1.465,−0.669	<0.001
Any	Any	−0.555	−1.870, 0.761	0.409
Cycling	Racquet sports			
None	None	*Ref*		
Any	None	−0.679	−1.174,−0.184	0.007
None	Any	−1.176	−1.599,−0.754	<0.001
Swimming	Aerobics			
None	None	*Ref*		
Any	None	−1.083	−1.473,−0.693	<0.001
None	Any	−0.742	−1.362,−0.122	0.019
Any	Any	−0.686	−1.720,0.347	0.193
Running	Aerobics			
None	None	*Ref*		
Any	None	−1.305	−1.729,−0.881	<0.001
None	Any	−1.784	−2.198,−1.370	<0.001
Any	Any	−1.973	−2.654,-1.292	<0.001
Running	American Football			
None	None	*Ref*		
Any	None	−3.951	−4.927,−2.975	<0.001
None	Any	−1.557	−1.935,−1.179	<0.001
Any	Any	−3.222	−4.416,-2.029	<0.001
Running	Racquet sports			
None	None	*Ref*		
Any	None	−0.433	−0.941, 0.076	0.096
None	Any	−1.516	−1.918,−1.115	<0.001
Any	Any	−1.672	−2.463,−0.882	<0.001

* Sex, BMI, race, current alcohol consumption, annual household income, intakes of energy, protein, carbohydrate, and fat, and the volume of other physical activity (MET, excluding the volume of the sports that were the main exposures in the corresponding model) were adjusted.

### Sensitivity analysis

Supplementary Table 3 shows the associations of specific types of physical activities with CVD risk in adults aged ≥ 30 years using multiple imputation dataset. The results of sensitivity analysis were consistent with the main results, except participation in swimming (β = −0.523, *95% CI*:−0.952,−0.094, *P* = 0.017). Furthermore, a more strong association of American Football with CVD risk was observed. Therefore, the results of sensitivity analysis were comparable with the main results.

## Discussion

This cross-sectional study from the NHANES was designed to evaluate the independent and interactional associations of specific types of physical activities with the 10-year CVD risk. The findings of this study implied that cycling, running, American Football, basketball, and aerobics were linked to a lower CVD risk. Furthermore, the most effective physical activity to decrease CVD risk was American Football. Significant associations with CVD risk were not observed for swimming and racquet sports. Furthermore, both participation in running and cycling, running and aerobics, and running and racquet sports was associated with a more decrease in the risk of CVD than only participation in one of the combination, which meant that there were synergistic associations of running and cycling, aerobics, and racquet sports with CVD risk. However, both participation in cycling and racquet sports, cycling and basketball, and swimming and aerobics was not associated with CVD risk compared to no participation in the combination, which meant that there were antagonistic associations of cycling and racquet sports and basketball, as well as swimming and aerobics with CVD risk.

Previous study declared that PA was the best prevention strategy to reduce the risk of CVD (20). PA can improve cardiovascular system, cardiac function, and insulin and lipoprotein metabolism (21). Meanwhile, regular physical exercise can improve endothelial function and lipid profile, and decrease inflammation markers and thrombosis (22). Therefore, there is a protective effect of PA on CVD. Randomized controlled trials reported that physical exercise can reduce the risk of CVD by decreasing adiposity, which is related to glucose control, metabolic dysfunction, and inflammation (23, 24). Previous study suggested that PA represents a natural and strong anti-inflammatory strategy in the prevention of CVD (25). Regular physical exercise can provoke marked increases in IL-6 and IL-10, which exert direct anti-inflammatory effects (26). Furthermore, PA can restrain secretion and production of TNF-α, which is involved in the pathogenesis of atherosclerosis and heart failure (27).

In the present study, cycling, American Football, basketball, and aerobics, but not swimming and racquet sports, were linked to a lower CVD risk. The presence of association of cycling with CVD risk was comparable with previous studies, including systematic review and meta-analysis (10, 28). It has been reported that walking and jogging can reduce the risk of CVD (29, 30). Previous studies found that participation in walking and aerobics was linked to a higher HDL-C level but a lower TG level, which were used to assess the risk of CVD in this study (31). Meanwhile, similar evidence was found that regular aerobic exercises have protective effects on SBP and can improve cardiorespiratory fitness (32, 33). Swimming was not associated with CVD risk in this study, which was consistent with a previous study (34). Another study found that participation in swimming was linked to lower risks of all-cause and CVD mortality (10). However, a review provided an inconclusive evidence on the association of swimming with all-cause and CVD mortality, and indicated that participation in football in leisure time can improve aerobics fitness and cardiovascular function and reduce adiposity, which supported the finding of this study (35). A previous study suggested that National Football League players had a significantly decreased CVD mortality (standardized mortality ratios: 0.68, *95% CI*: 0.56- 0.81) (36), which partly supported the finding of this study. Meanwhile, defensive linemen but not offensive linemen had an increased CVD mortality, which implied that different football player positions can influence the risk of CVD (36). However, since no data of football player positions was collected in the NHANES, it need further be illustrated in the future. Participation in racquet sports was not associated with CVD risk in this study, which might be contradictory with previous study (10). The possible reasons were the different elements used to define racquet sports. In a previous study, racquet sports included badminton, tennis, and squash. However, according to the involved types of sports in the NHANES, racquet sports included baseball, golf, hockey, racquetball, softball, and tennis in this study. A prospective cohort study showed that participation in tennis but not racquetball can decrease CVD risk (37). To date, little comparable data on the association of basketball with CVD risk are available. There was only one study to investigate the association of basketball with the risk of CVD, indicating an insignificant association (34). Nevertheless, the study participants limited to subjects aged 45–64 years.

A key finding of this study was that there were interactions between different types of physical activities, including synergistic and antagonistic associations. A previous study reported that there is a synergistic association of resistance training and aerobic exercise with the vascular endothelial function (38). In recent years, health benefits of the accumulation of physical activities have been reported (11, 12, 39), However, the underlying mechanisms on the interplay between different types of physical activities were not well known, and need further evidence from laboratory in the future.

### Strengths and limitations

A notable strength of this study was that the interactions between different types of physical activities were assessed and found the potential synergistic and antagonistic associations. Therefore, this study contributes to the existing body of knowledge suggesting that it is critical to choose proper types of physical activities and the combination for health benefits. However, there were a few limitations. Firstly, this study limited to a cross-sectional design, which cannot confirm the causal associations of specific types of physical activities with CVD risk. Secondly, specific types of physical activities participated by each subject might alter over time. However, the longitudinal changes in participation behavior cannot be considered in this study. Thirdly, a limited number of specific types of sports were considered in this study, which limits the number of comparisons we could make.

In conclusion, the significant associations of participation in certain types of physical activities with CVD risk were found, indicating substantial reductions in CVD risk for cycling, American Football, basketball, running, and aerobics. Furthermore, if subjects participated in two or more types of physical activities, there might be synergistic or antagonistic associations of multiple types of physical activities with the risk of CVD. It seems therefore that the findings of this study contribute to the body of evidence supporting the protective effects of physical activities on CVD. Meanwhile, the sport community should make health-enhancing sport packages to promote public health according to the interplay between the different types of physical activities.

## Data availability statement

Publicly available datasets were analyzed in this study. This data can be found here: https://www.cdc.gov/nchs/index.htm.

## Ethics statement

The studies involving human participants were reviewed and approved by the NHANES Institutional Review Board (1999-2002: Protocol #98-12). The patients/participants provided their written informed consent to participate in this study.

## Author contributions

CD contributed to conception and design of the study. QW and XW organized the database. LW, PM, and FW performed the statistical analysis and interpreted the results. BH wrote the first draft of the manuscript. All authors contributed to manuscript revision, read, and approved the submitted version.

## Conflict of interest

The authors declare that the research was conducted in the absence of any commercial or financial relationships that could be construed as a potential conflict of interest.

## Publisher's note

All claims expressed in this article are solely those of the authors and do not necessarily represent those of their affiliated organizations, or those of the publisher, the editors and the reviewers. Any product that may be evaluated in this article, or claim that may be made by its manufacturer, is not guaranteed or endorsed by the publisher.
