# Supplementary material for: Associations of specific types of physical activities with 10-year risk of cardiovascular disease among adults: Data from the national health and nutrition examination survey 1999–2006
Source: Front Public Health. 2022 Jul 25;10:964862. doi: 10.3389/fpubh.2022.964862 (PMC9358206; doi:10.3389/fpubh.2022.964862)
Supplement: Supplementary file 1 [file Data_Sheet_1.docx]

**Supplementary Material**

Supplementary Table 1 Sex-specific distributions of duration (minutes/month) and energy expenditure weighted volume (MET-hours/month) of specific types of physical activities from the participators in each sport only

|  | Duration (minutes/month) | | Volume (MET-hours/month) | | Intensity | |
| --- | --- | --- | --- | --- | --- | --- |
|  | Median | IQR (P_25_, P_75_) | Median | IQR (P_25_, P_75_) | Moderate (n (%)) | Vigorous (n (%)) |
| Cycling |  |  |  |  |  |  |
| Male | 240 | 100-540 | 8.0 | 4.0-8.0 | 210(33.49) | 417(66.51) |
| Female | 240 | 90-450 | 8.0 | 4.0-8.0 | 170(38.90) | 267(61.10) |
| Swimming |  |  |  |  |  |  |
| Male | 167.5 | 60-465 | 8.0 | 6.0-8.0 | 77(35.65) | 139(64.35) |
| Female | 240 | 90-540 | 6.0 | 6.0-8.0 | 130(51.18) | 124(48.82) |
| Running |  |  |  |  |  |  |
| Male | 270 | 120-585 | 7.0 | 7.0-10.0 | 111(14.98) | 630(85.02) |
| Female | 300 | 120-520 | 7.0 | 7.0-10.0 | 144(23.41) | 471(76.59) |
| American Football |  |  |  |  |  |  |
| Male | 240 | 120-560 | 10.0 | 8.0-10.0 | 37(22.56) | 127(77.44) |
| Female | 90 | 42.5-270 | 8.0 | 5.5-10.0 | 13(46.43) | 15(53.57) |
| Basketball |  |  |  |  |  |  |
| Male | 240 | 90-480 | 8.0 | 8.0-8.0 | 59(19.93) | 237(80.07) |
| Female | 120 | 60-300 | 8.0 | 6.0-8.0 | 22(40.74) | 32(59.26) |
| Racquet sports |  |  |  |  |  |  |
| Male | 720 | 240-1530 | 3.5 | 3.5-6.0 | 448(76.84) | 135(23.16) |
| Female | 360 | 180-810 | 5.0 | 3.5-7.0 | 113(62.43) | 68(37.57) |
| Aerobics |  |  |  |  |  |  |
| Male | 255 | 120-540 | 4.5 | 4.5-7.0 | 308(61.85) | 190(38.15) |
| Female | 300 | 130-600 | 5.0 | 4.5-7.0 | 526(57.30) | 392(42.70) |

Supplementary Table 2 The interactions between the different types of physical activities on the risk of CVD^※^

| Types of sports | Cycling | Swimming | Running | American Football | Basketball | Racquet sports | Aerobics |
| --- | --- | --- | --- | --- | --- | --- | --- |
| Cycling | - | 0.789 | 0.016 | 0.095 | 0.002 | 0.002 | 0.469 |
| Swimming |  | - | 0.175 | 0.236 | 0.507 | 0.624 | 0.041 |
| Running |  |  | - | 0.009 | 0.044 | 0.972 | 0.019 |
| American Football |  |  |  | - | 0.083 | 0.877 | 0.213 |
| Basketball |  |  |  |  | - | 0.536 | 0.480 |
| Racquet sports |  |  |  |  |  | - | 0.822 |
| Aerobics |  |  |  |  |  |  | - |

^※^Sex, BMI, race, current alcohol consumption, annual household income, intakes of energy, protein, carbohydrate, and fat, and the volume of other physical activity (MET, excluding the volume of the sports that were the main exposures in the corresponding model) were adjusted.

Supplementary Table 3 Associations of specific types of physical activities with CVD risk in adults aged ≥ 30 years using multiple imputation dataset^※^

|  | *β* | *95% CI* | *P* |
| --- | --- | --- | --- |
| Cycling |  |  |  |
| None | *Ref* |  |  |
| Any | -1.045 | -1.358,-0.733 | <0.001 |
| Swimming |  |  |  |
| None | *Ref* |  |  |
| Any | -0.523 | -0.952,-0.094 | 0.017 |
| Running |  |  |  |
| None | *Ref* |  |  |
| Any | -1.530 | -1.821,-1.239 | <0.001 |
| American Football |  |  |  |
| None | *Ref* |  |  |
| Any | -2.976 | -3.699,-2.253 | <0.001 |
| Basketball |  |  |  |
| None | *Ref* |  |  |
| Any | -2.219 | -2.753,-1.685 | <0.001 |
| Racquet sports |  |  |  |
| None | *Ref* |  |  |
| Any | -0.292 | -0.644,0.059 | 0.103 |
| Aerobics |  |  |  |
| None | *Ref* |  |  |
| Any | -1.101 | -1.388,-0.815 | <0.001 |

^※^Sex, BMI, race, current alcohol consumption, annual household income, intakes of energy, protein, carbohydrate, and fat, and the volume of other physical activity (MET, excluding the volume of the sport that was the main exposure in the corresponding model) were adjusted.
